# I see you? - Predicting who will require intensive care

**DOI:** 10.1186/1757-7241-21-S2-A29

**Published:** 2013-09-09

**Authors:** Maria Bjørn Marcussen, Christian Backer Mogensen

**Affiliations:** 1 OUH Odense, Sygehus Lillebælt, Kolding, Region of Southern Denmark, Denmark

## Background

Delayed transfer to the intensive care unit (ICU) is correlated with higher morbidity and mortality in emergency department (ED) patients. If it is possible to identify patients at risk of transfer to the ICU shortly after arrival to the ED, early goal-directed therapy could theoretically reduce the incidence of ICU transfer and improve outcome for the critically ill. No widely used scoring system exists for identifying these patients in the heterogeneous population of the ED. The aim of this study was to identify possible predictors obtainable on admission for ICU therapy.

## Methods

We conducted a retrospective case-control study with a total of 10.000 acute patients. The case group consisted of adult patients, transferred to the ICU between 3 and 36 hours after arrival, whom had blood gas analysis done. The specialty-matched control group consisted of adult patients who had blood gas analysis done but were not transferred to the ICU. A total of 325 patients, 125 cases and 250 controls, were compared with regards to vital parameters, age, gender, blood gas parameters, GCS, Charlson comorbidity index, tobacco- and alcoholconsumption and number of prescription drugs. We performed uni- and multivariate regression analyses to identify risk factors associated with later ICU transfer. A p-value below 0.05 was considered statistically significant.

## Results

We found age between 60 and 80 years (OR 3.2), systolic blood pressure below 90 or diastolic blood pressure below 50 (OR 3.7), oxygen saturation below 90 (OR 3.2), temperature below 36 (OR 4.1), Charlson score more than 0 (OR 3.1), lactate above 4 (OR 5.3), pH below 7.35 (OR 8.4) and pCO2 above 6.3 (OR 5.4) on admission to be significantly associated with later ICU transfer. At multivariate analysis a pH value less than 7.36 (OR 14.4), oxygen saturation below 90 and low blood pressure (OR 4.4) remained significantly associated with ICU transfer.

## Conclusion

These parameters can be used as part of ED triage identifying high-risk patients who could benefit from early anesthesiologist consult, early goal directed therapy or simply closer observation.

